# Non-fasting versus fasting before percutaneous cardiac procedures: a systematic review and meta-analysis of randomized controlled trials

**DOI:** 10.1186/s13741-024-00485-6

**Published:** 2025-02-28

**Authors:** Elsayed Balbaa, Ahmed A. Ibrahim, Mohammad Bazzazeh, Shehroze Tabassum, Shrouk Ramadan, Ahmed Farid Gadelmawla, Abdelrahman Elshimy, Obieda Altobaishat, Mohamed Abuelazm

**Affiliations:** 1 Faculty of Medicine, Alexandria University, Alexandria, Egypt; 2 Faculty of Medicine, Menoufia University, Menoufia, Egypt; 3 Department of Medicine, King Edward Medical University, Lahore, Pakistan; 4 Faculty of Medicine, Ain Shams University, Cairo, Egypt; 5 Faculty of Medicine, Jordan University of Science and Technology, Irbid, Jordan; 6 Faculty of Medicine, Tanta University, Tanta, Egypt

**Keywords:** Aspiration, Fasting, Nausea, Percutaneous cardiac catheterization, Patient satisfaction, Vomiting

## Abstract

**Background and objective:**

Despite the absence of scientific evidence, fasting before percutaneous cardiac catheterization is still recommended to minimize complications. This systematic review and meta-analysis aimed to compare the outcomes of non-fasting protocols in patients undergoing percutaneous cardiac procedures.

**Materials and methods:**

A systematic search of PubMed, Scopus, WOS, Embase, and Cochrane was conducted until September 2024. Dichotomous outcomes were pooled using risk ratio (RR), while continuous outcomes were pooled using standardized mean difference (SMD). PROSPERO ID: CRD42024586147.

**Results:**

Five RCTs with 2034 patients were included. There was no significant difference between both groups regarding patient satisfaction score [SMD − 0.65, 95% CI (− 1.39, 0.09), *P* = 0.08], intra/postoperative aspiration (RR 1.00, 95% CI [0.20, 4.96], *P* = 1.00), postprocedural pneumonia (RR 0.60, 95% CI [0.14, 2.51], *P* = 0.49), emergency endotracheal intubation (RR 0.99, 95% CI [0.10, 9.51], *P* = 1.00), nausea/vomiting (RR 0.89, 95% CI [0.46, 1.76], *P* = 0.75), anti-emetic use (RR 0.49, 95% CI [0.24, 1.03], *P* = 0.06), hypoglycemia (RR 0.74, 95% CI [0.43, 1.28], *P* = 0.28), and the need for inotrope/vasopressor therapy (RR 1.03, 95% CI [0.81, 1.30], *P* = 0.82). However, the non-fasting approach significantly decreased the sensation of tiredness/fatigue (SMD − 0.31 with 95% CI [− 0.51, − 0.11], *P* < 0.001).

**Conclusion:**

The non-fasting protocol demonstrated comparable efficacy, safety, and overall satisfaction to the conventional fasting approach.

**Review registration:**

PROSPERO CRD42024586147.

**Supplementary Information:**

The online version contains supplementary material available at 10.1186/s13741-024-00485-6.

## Introduction

Minimally invasive cardiac procedures (MICPs) have revolutionized cardiac care by offering less invasive and more patient-friendly alternatives to traditional open-heart surgery. These often result in faster and better cosmetic outcomes (Teman et al. 2021; Park 1999; Abdelazeem et al. 2022). Although these procedures are becoming more prevalent, the ideal preprocedural fasting protocol is still being debated (Park 1999; Pimenta and Aguilar-Nascimento 2014). The fasting protocols initially involved no food or drink from the night before the procedures. This prolonged fasting is thought to reduce the risk of vomiting, aspiration, and death (Maltby 2006). Traditional protocols currently involve fasting 6–8 h before the procedure (Pimenta and Aguilar-Nascimento 2014). The American Society of Anesthesiologists recommended shortening fasting protocols for healthy patients undergoing elective procedures. They can consume clear liquids for up to 2 h and solid food for up to 6 h before surgery (Practice Guidelines for Preoperative Fasting and the Use of Pharmacologic Agents to Reduce the Risk of Pulmonary Aspiration: Application to Healthy Patients Undergoing Elective Procedures 2017). However, there is a significant variation in the followed fasting protocols (Rolley et al. 2015).

Furthermore, the duration of fasting is often prolonged (Abdullah Al Maqbali 2016). This could lead to hypoglycemia, dehydration, impaired metabolism, and an increase in the risk of vasovagal attacks (Hamid et al. 2014; Rolley et al. 2015; Yang et al. 2017). Recently, it has been shown that non-fasting could be a promising approach. This approach could lead to favorable outcomes without compromising patient safety (Choi et al. 2023; Noba and Wakefield 2019). Non-fasting protocols allow patients to have light meals or fluids closer to the procedure time. Patients following this approach reported higher overall well-being and satisfaction scores without an increase in adverse events (Bode et al. 2022; Boukantar et al. 2024; He et al. 2022; Power et al. 2012). Despite the growing evidence in favor of non-fasting protocols, the optimal fasting approach is still unclear; it may vary depending on the type of procedure and patient factors (Practice Guidelines for Preoperative Fasting and the Use of Pharmacologic Agents to Reduce the Risk of Pulmonary Aspiration: Application to Healthy Patients Undergoing Elective Procedures 2017). Considering the importance of determining the optimal preoperative fasting protocol, we conducted this systematic review and meta-analysis to investigate the effects of non-fasting protocols in patients undergoing MICPs.

## Methodology

### Protocol registration

This systematic review and meta-analysis followed the PRISMA (Preferred Reporting Items for Systematic Reviews and Meta-Analyses) statement (Page et al. 2020) and the Cochrane Handbook for Systematic Reviews and Meta-Analyses guidelines (Cochrane Handbook for Systematic Reviews of Interventions 2024). This review’s protocol has been published and registered in PROSPERO under the ID (CRD42024586147).

### Data sources and search strategy

The Cochrane Central Register of Controlled Trials (CENTRAL), PubMed (MEDLINE), Web of Science (WoS), SCOPUS, and EMBASE databases were all searched until September 2024. The results of each database’s search terms and keywords are shown in Table S1.

### Eligibility criteria

We used the Population, Intervention, Comparison, and Outcomes (PICO) criteria to select eligible randomized controlled trials (RCTs): population (patients undergoing MICPs, including percutaneous coronary intervention (PCI), transcatheter aortic valve replacement (TAVR), and catheter ablation); intervention (non-fasting); comparison (fasting); and outcomes: the primary outcome was the composite satisfaction score, whereas the secondary outcomes included hunger, thirst, anxiety, tiredness/fatigue, intra/postop aspiration, postprocedural pneumonia, emergency endotracheal intubation, nausea/vomiting, anti-emetic use, hypoglycemia (≤ 0.7 g/L), intraoperative fluid provision, contrast-induced acute kidney injury (AKI), need for inotrope/vasopressor therapy, and need for ventilation.

### Study selection

We conducted the review via the Covidence online tool. After eliminating duplicates, two authors (M.B. and S.R.) evaluated each record they retrieved separately. Two authors (O.A. and A.E.) reviewed the complete texts of the records for the first full-text screening for eligibility criteria. All differences were settled by consensus after consulting (M.A.).

### Data extraction

The baseline characteristics and outcomes data were extracted using a Microsoft Excel extraction sheet (M.B. and S.R.), and the senior author (M.A.) settled disagreements. These data were arranged as follows: (1) study characteristics, such as study ID, study design, country, anesthesia modality, non-fasting protocol, fasting protocol, fasting time, types of cardiac procedures (%), and primary endpoints; (2) baseline patient characteristics, including the number of patients in each group, age, sex, hypertension, diabetes mellitus, and gastroesophageal reflux disease; and (3) the previously mentioned outcome measures.

### Risk of bias

The Cochrane Risk of Bias 2 (RoB 2) tool (Sterne et al. 2019) was utilized by two reviewers (A.E. and S.R.) to assess the overall quality of the included RCTs. Each of the six domains comprising the RoB 2 tool focuses on a specific aspect of trial conduct, design, and reporting. (1) randomization procedure; (2) deviations from intended interventions; (3) missing outcome data; (4) outcome measurement; (5) reporting result selection; and (6) overall bias. Conflicts were resolved through discussions with the senior author (M.A.).

### Statistical analysis

The study employed R version 4.3, utilizing the “meta”, “metafor,” and “dmetar” packages for statistical analysis. The analysis combined results from multiple studies using either risk ratios (for dichotomous outcomes) or mean differences (for continuous outcomes), both with 95% confidence intervals. A random-effects model was applied when significant heterogeneity was detected via the chi-square test and *I*^2^ statistic; otherwise, a fixed-effect model was used. Heterogeneity was interpreted according to the Cochrane Handbook (chapter nine) (Cochrane Handbook for Systematic Reviews of Interventions 2024), with an *I*^2^ value of 0–40% indicating low heterogeneity, 30–60% indicating moderate heterogeneity, 50–90% possibly representing substantial heterogeneity, and 75–100% signifying considerable heterogeneity. A chi-square test *p*-value less than 0.1 was considered statistically significant for heterogeneity.

## Results

### Search results and study selection

**Fig. 1:**
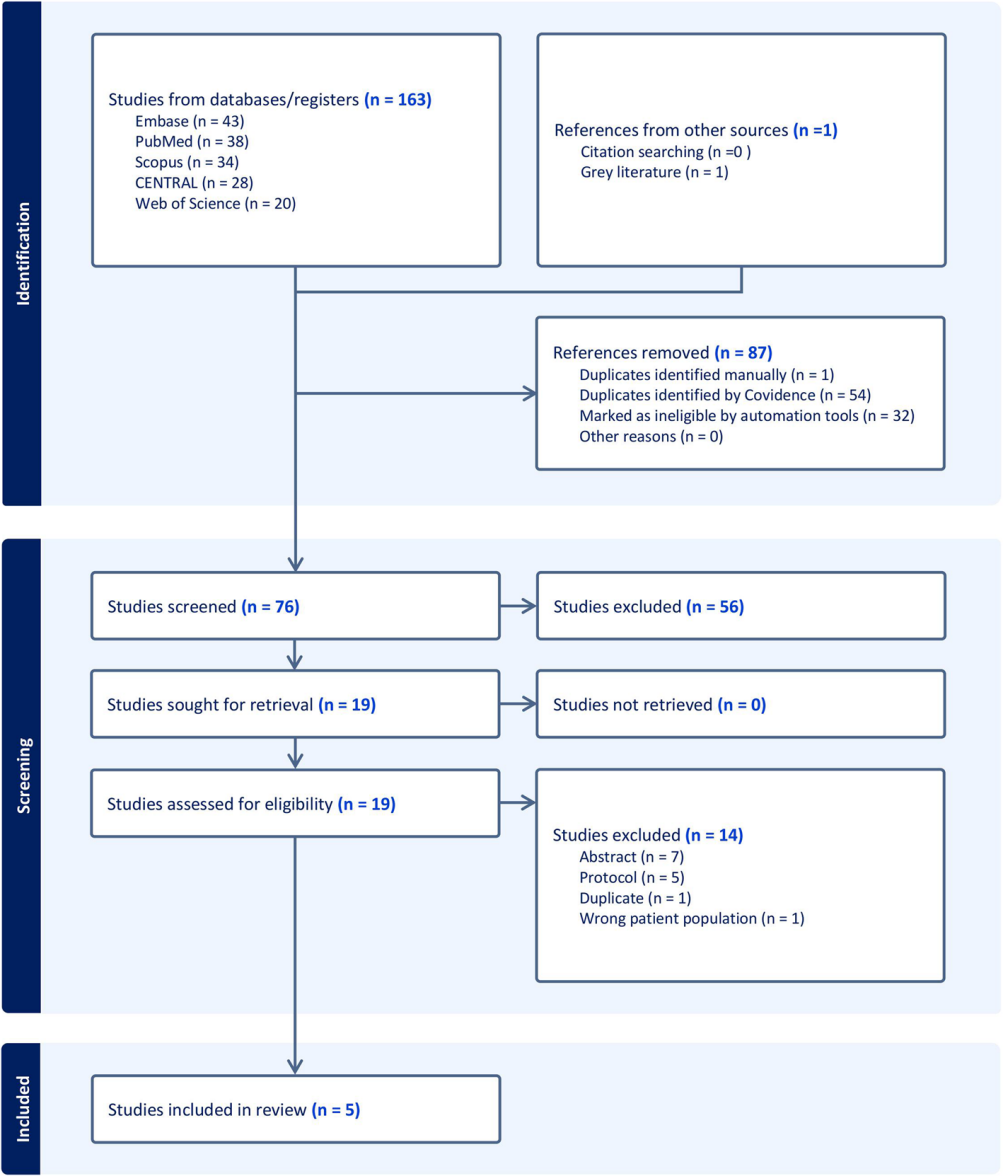
PRISMA flow chart of the screening process

By searching databases, we retrieved 164 records, and 87 references were excluded by Covidence, leaving 76 references for primary screening by title and abstract. After screening by title and abstract, 19 articles were available to be assessed in full-text screening. Finally, we included five studies with 2034 patients in this systematic review and meta-analysis. The PRISMA flow chart of the selection process is shown in Fig. 1.

### Characteristics of the included studies

All the included studies were single-center RCTs; four were single-blinded (Bode et al. 2022; Boukantar et al. 2024; Ferreira et al. 2024; Woods et al. 2024), and the other was open-label (Atkinson et al. 2023). Three studies were conducted in the USA, one in France, and another in Germany. The summary of the included studies and the baseline characteristics are presented in Tables 1 and 2, respectively.

**Table 1:** Summary characteristics of the included RCTs

Study ID	Country	Design	Sample size	Anesthesia modality	Non-fasting protocol	Fasting protocol	Fasting time	Types of cardiac procedures (%)	Primary endpoints
Atkinson et al. 2023	USA	Single-center unblinded RCT	181	The patient was prescribed short-acting narcotics and benzodiazepine in combination with a dexmedetomidine or propofol infusion, titrated to a RASS of − 3 to − 4	Patients could consume as much fluid as they wanted up to 2 h before the procedure	Before the surgery, the group was told to fast after midnight save for a drink of water with drugs	2 h	Non-fasting: TAVR (23.3) Arrhythmia ablation (76.7) Fasting: TAVR (26.4) Arrhythmia ablation (73.6)	Patients self-reported feelings of thirst, hunger, headache, nausea, dizziness, and anxiety in the satisfaction survey
Bode et al. 2022	Germany	Single-center, single-blinded RCT	201	The operation site received local anaesthesia with 20–40 mL Mepivacaine 1%, with additional intravenous analgesics and sedatives, such as fentanyl, midazolam, or Propofol, as needed	Before the CIED operation, patients could eat and drink for up to 1 h	Fasting for at least 6 h for solids and 2 h for drinks before the CIED treatment	2 h (drinks), 6 h (food)	Fasting and non-fasting: CARDIAC implantable electronic devices (100)	Procedural patients’ well-being scores
Woods et al. 2024	USA	Single-center, single-blinded RCT	197	NA	Patients could follow a low-fat, cholesterol, salt, and acidity diet until the operation	Patients were only allowed to consume water with medicine after midnight until the scheduled surgery	6 h	Fasting and non-fasting: Angiographies (57.4) Angioplasties (42.6)	Patient-reported satisfaction and complications
Boukantar et al. 2024	France	Single-center, single-blinded RCT	739	Local anesthesia using subcutaneous lidocaine 1% (1 to 20 mL) and moderate sedation with IV midazolam	Patients could eat and drink whenever they wanted	Patients were instructed not to consume solid meals or drinks for at least 6 h	NA	Non-fasting: Angiographies (67.3) Angioplasties (9.3) Others (23.4) Fasting: Angiographies (69.7) Angioplasties (8.7) Others (21.6)	Vasovagal reaction, hypoglycemia, isolated nausea, and vomiting
Ferreira et al. 2024	USA	Single-center, single-blinded RCT	716	Midazolam with mean dose 1.12 mg Fentanyl with a mean dose of 40 µg	No fasting, regular meals suggested	Six hours of fasting for solid food and 2 h for clear fluids	12 h for solids, 7 h for liquid	Non-fasting: Coronary procedure (83) Cardiac implantable electronic devices (17) Fasting: Coronary procedure (85.5) Cardiac implantable electronic devices (14.5)	Aspiration Pneumonia, hypotension, hypertension, and hyperglycemia

*Abbreviations: CIED* cardiac implantable electronic devices, *NA* not available, *RASS* renin–angiotensin–aldosterone system, *RCT* randomized clinical trial, *TAVR* transcatheter aortic valve replacement

**Table 2:** Baseline characteristics of the participants

Study ID	Groups	Sample size	Age, mean (SD)	Sex, Female *N*, (%)	HTN *N*, (%)	DM *N* (%)	GERD ***N*, (%)**
Atkinson et al. 2023	Non-fasting	90	68.1 (11.8)	32 (35.56)	66 (73.3)	21 (23.3)	23 (25.6)
	Fasting	91	68.9 (11.5)	28 (30.77)	68 (74.4)	20 (22)	25 (27.5)
Bode et al. 2022	Non-fasting	100	71.6 (13.2)	33 (33)	72 (72)	40 (40)	1 (1)
	Fasting	101	72.5 (9.8)	33 (32.7)	78 (77.2)	39 (38.6)	4 (4)
Woods et al. 2024	Non-fasting	100	62.7 (12.7)	74 (74)	NA	NA	NA
	Fasting	97	62.7 (12.7)	74 (76.3)	NA	NA	NA
Boukantar et al. 2024	Non-fasting	376	68 (11)	94 (25)	270 (72)	113 (30)	NA
	Fasting	379	67 (12)	92 (24.3)	273 (73)	106 (28)	NA
Ferreira et al. 2024	Non-fasting	358	69 (10.9)	122 (34.1)	249 (69.9)	95 (26.5)	NA
	Fasting	358	70 (11.4)	127 (35.5)	250 (69.8)	97 (27.1)	NA

*Abbreviations: DM* Diabetes mellitus, *GERD* gastroesophageal reflux disease, *HTN* hypertension, *N* number, *NA* not available, *SD* standard deviation

### Risk of bias

**Fig. 2:**
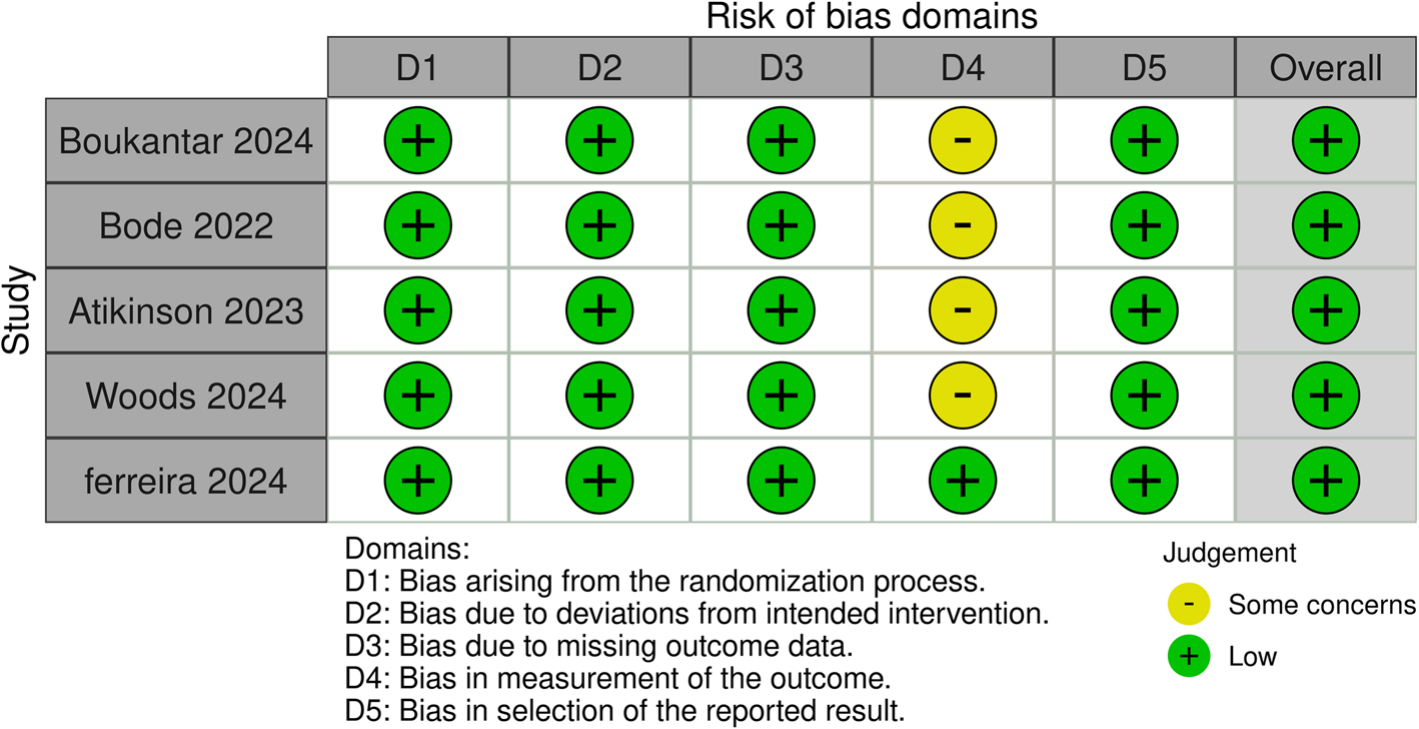
Quality assessment of the risk of bias in the included trials. The upper panel presents a schematic representation of risks (low = green, unclear = yellow, and high = red) for specific types of biases of each study in the review. The lower panel presents risks (low = green, unclear = yellow, and high = red) for the subtypes of biases of the combination of studies included in this review

All the included studies had an overall low risk of bias. No biases were detected regarding the selection (randomization) process, such as random sequence generation and concealment of allocators. No study had limited reporting of any critical outcomes. All trials analyzed patients via intention-to-treat analysis to address the lack of outcome data. There were some concerns in measuring the outcomes, as it was unclear whether the outcome assessors were blinded, and our outcomes were mainly subjectively measured (Fig. 2).

### Meta-analysis

#### Primary outcome: composite satisfaction score

**Fig. 3:**
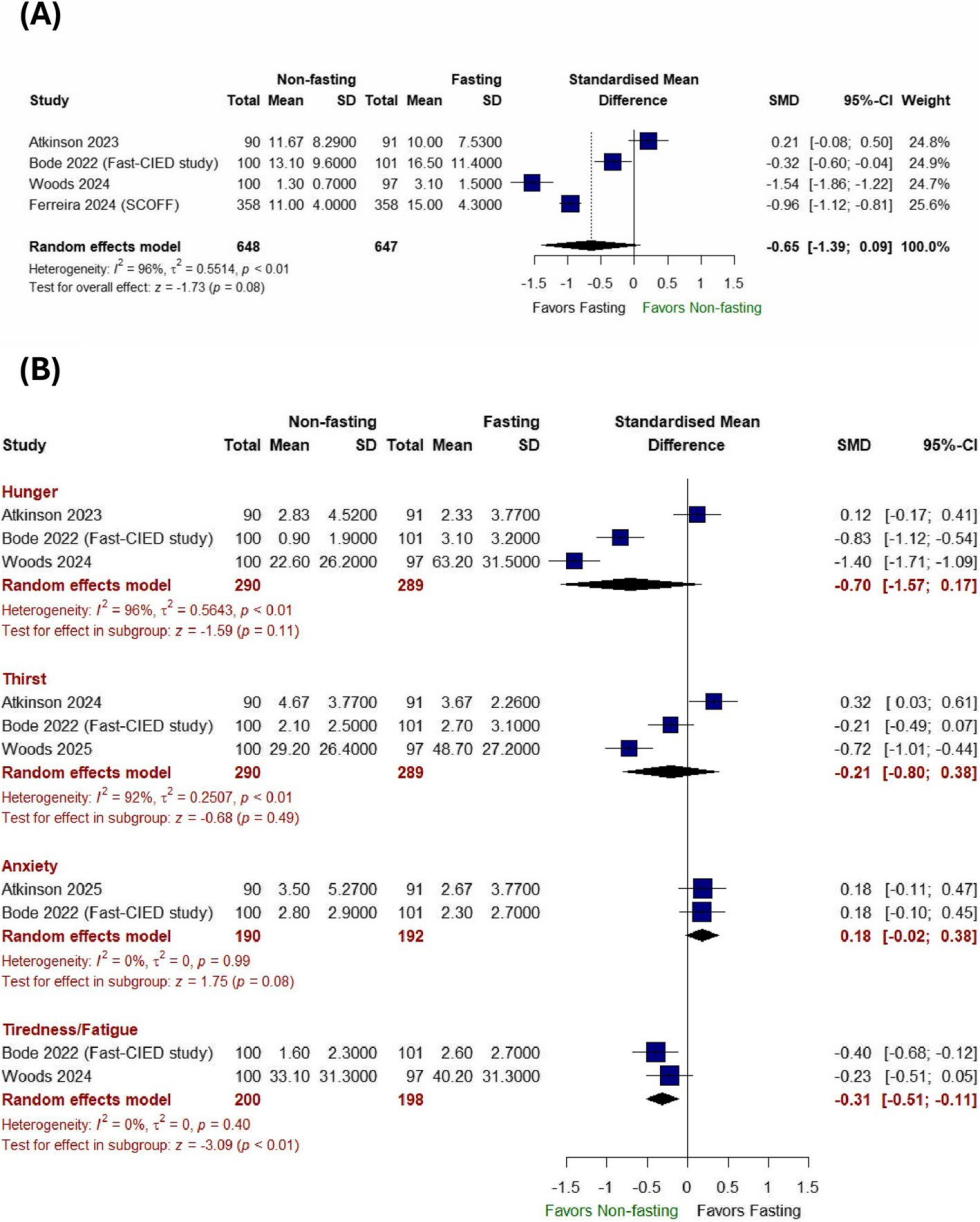
Forest plot of composite satisfaction score, hunger, thirst, anxiety, and tiredness/fatigue; RR: risk ratio; CI: confidence interval

There was no significant difference in the composite satisfaction score between the non-fasting and fasting approaches (SMD − 0.65 with 95% CI [− 1.39, 0.09], *P* = 0.08) (Fig. 3A). The pooled studies were heterogeneous (*I*^2^ = 96%, *P* < 0.01). Sensitivity analysis did not resolve the heterogeneity (Figure S1).

#### Secondary outcomes

##### Specific satisfaction outcomes

There was no significant difference between the non-fasting and fasting approaches in terms of the sensations of hunger (SMD: − 0.70 with 95% CI [− 1.57, 0.17], *P* = 0.11), thirst (SMD: − 0.21 with 95% CI [− 0.80, 0.38], *P* = 0.49), or anxiety (SMD 0.18 with 95% CI [− 0.02, 0.38], *P* = 0.08). However, the non-fasting approach significantly decreased the sensation of tiredness/fatigue (SMD − 0.31 with 95% CI [− 0.51, − 0.11], *P* < 0.001) (Fig. 3B).

The pooled studies were homogeneous regarding anxiety (*I*^2^ = 0%, *P* = 0.99) and tiredness/fatigue (*I*^2^ = 0%, *P* = 0.40). However, pooled studies were heterogeneous in hunger (*I*^2^ = 96%, *P* < 0.001) and thirst (*I*^2^ = 92%, *P* < 0.001). Sensitivity analysis did not resolve the heterogeneity (Figures S2 and S3).

##### Periprocedural outcomes

There was no significant difference in the length of hospital stay (hours) (MD − 1.28 with 95% CI [− 2.60, 5.16], *P* = 0.52) **(**Fig. S4), postoperative creatinine level (mg/dl) (MD − 0.06 with 95% CI [− 0.18, 0.07], *P* = 0.39) (Fig. S5), heart rate at the start of the procedure (MD: 0.91 with 95% CI [− 0.80, 2.62], *P* = 0.30) (Fig. S6), or mean arterial blood pressure at the start of the procedure (MD − 1.27 with 95% CI [− 3.34, 0.80], *P* = 0.23) (Fig. S6).

Pooled studies were homogeneous in length of hospital stay (*I*^2^ = 0%, *P* = 0.38), heart rate at the start of the procedure (*I*^2^ = 0%, *P* = 0.99), and mean arterial blood pressure at the start of the procedure (*I*^2^ = 0%, *P* = 0.40). However, pooled studies were heterogeneous regarding postoperative creatinine levels (*I*^2^ = 62%, *P* = 0.10). Sensitivity analysis did not resolve the heterogeneity (Fig. S5).

##### Safety outcomes

**Fig. 4:**
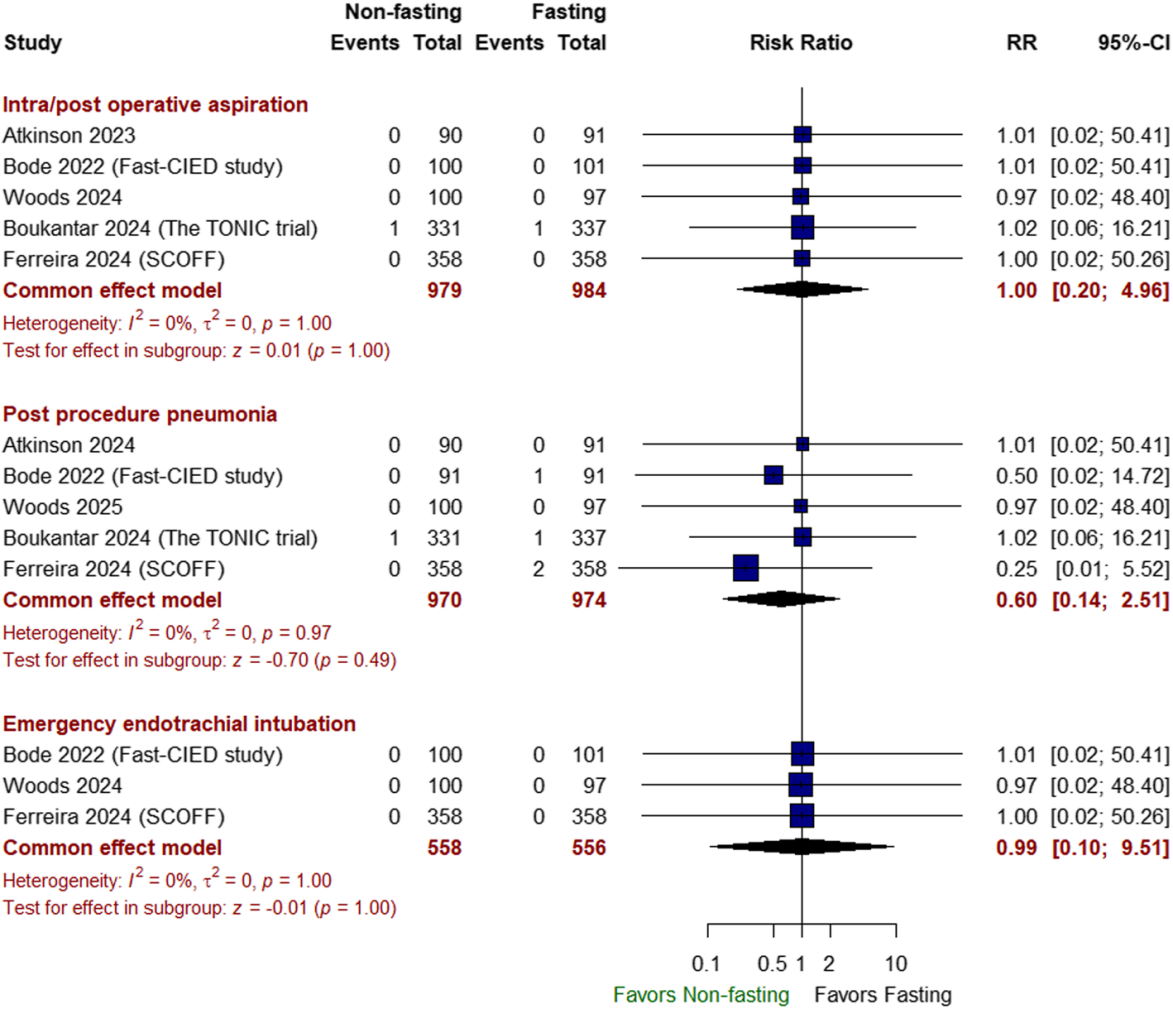
Forest plot of intra/postoperative aspiration, postprocedural pneumonia, and emergency endotracheal intubation; RR: risk ratio; CI: confidence interval

**Fig. 5:**
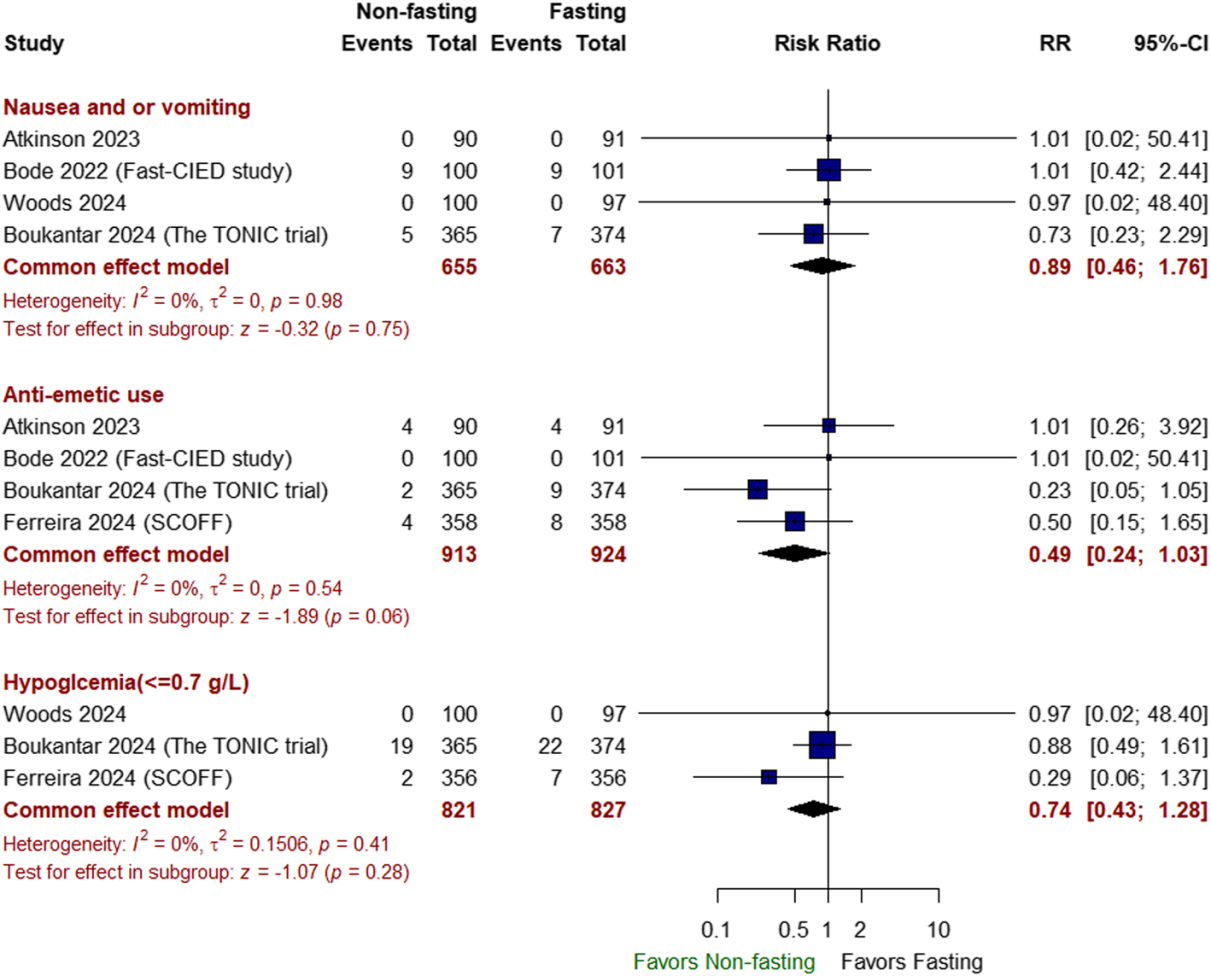
Forest plot of nausea and/or vomiting, anti-emetic use, hypoglycemia (≤ 0.7 g/L), RR: risk ratio, CI: confidence interval

**Fig. 6:**
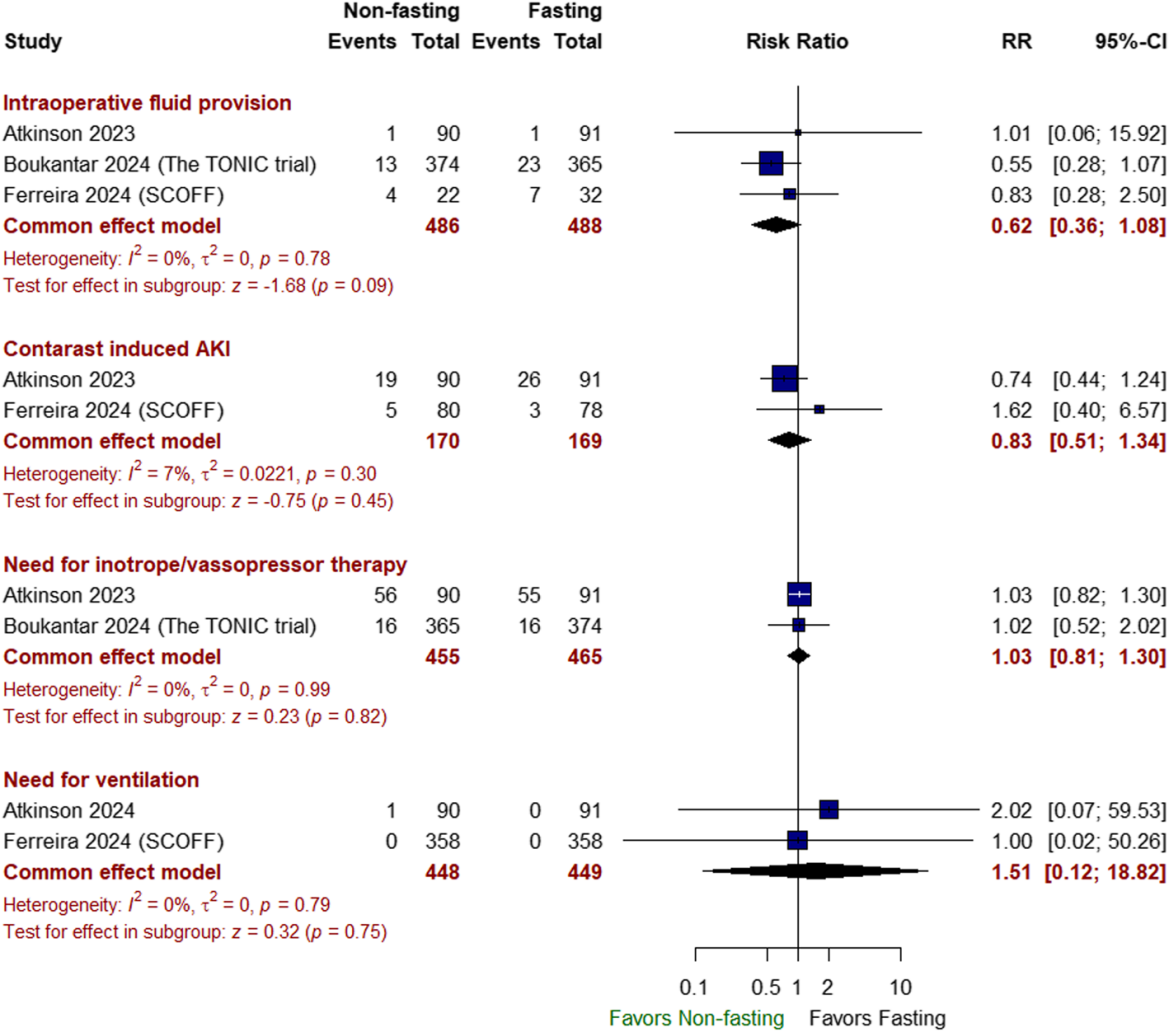
Forest plot of (intraoperative fluid provision, contrast-induced AKI, need for inotrope/vasopressor therapy, need for ventilation), RR: risk ratio, CI: confidence interval

There was no significant difference between non-fasting and fasting approaches in terms of the incidence of intra/postoperative aspiration (RR 1.00 with 95% CI [0.20, 4.96], *P* = 1.00), postprocedural pneumonia (RR 0.60 with 95% CI [0.14, 2.51], *P* = 0.49), emergency endotracheal intubation (RR 0.99 with 95% CI [0.10, 9.51], *P* = 1.00) (Fig. 4), nausea/vomiting (RR 0.89 with 95% CI [0.46, 1.76], *P* = 0.75), anti-emetic use (RR: 0.49 with 95% CI [0.24, 1.03], *P* = 0.06), hypoglycemia (RR 0.74 with 95% CI [0.43, 1.28], *P* = 0.28) (Fig. 5), intraoperative fluid provision (RR 0.62 with 95% CI [0.36, 1.08], *P* = 0.09), contrast-induced AKI (RR 0.83 with 95% CI [0.51, 1.34], *P* = 0.45), need for inotrope/vasopressor therapy (RR 1.03 with 95% CI [0.81, 1.30], *P* = 0.82), and need for ventilation (RR 1.51 with 95% CI [0.12, 18.82], *P* = 0.75) (Fig. 6).

The pooled studies were homogeneous in all the previously mentioned outcomes (*I*^2^ < 50%, *P* > 0.1).

## Discussion

This systematic review and meta-analysis, which included five RCTs, evaluated the latest evidence comparing non-fasting and traditional fasting protocols in 2034 patients who underwent MICPs. Our principal results highlight that the non-fasting protocol offers outcomes comparable to the conventional fasting approach, with the added advantage of reducing fatigue.

Fasting is broadly implemented owing to perceived risk and theoretical considerations. The 2021 American Heart Association scientific statement on evidence-based recommendations in cardiac catheterization recognizes the unclear benefit of prolonged preprocedural fasting, referring to evidence for this being “weak,” and emphasizes a need to define best practices (Bangalore et al. 2021). Furthermore, the detrimental effects of prolonged nil per os (NPO) status, including patient dissatisfaction and disruption of homeostasis, particularly in those on diabetic medication and individuals with renal insufficiency, are well documented in medical literature. Interestingly, our pooled analysis revealed no significant difference in the composite satisfaction score between the non-fasting and fasting approaches.

Concerning specific satisfaction outcomes, there were no significant differences in sensations of hunger, thirst, or anxiety; however, the non-fasting approach significantly reduced feelings of tiredness and fatigue, which holds particular importance for older adults. This underscores the non-inferiority of non-fasting compared with fasting concerning patient satisfaction. The current body of evidence supports our findings. For example, a prior study by Mishra et al. (2019) revealed that non-fasting is associated with improved patient satisfaction compared with traditional fasting practices.

In terms of efficacy outcomes, the non-fasting approach did not significantly differ from the traditional fasting approach in terms of hospital stay duration, postoperative creatinine levels, heart rate, or mean blood pressure at the start of the procedure in our analysis. It is well established that postponing or canceling procedures adds to the burden on the healthcare system, often leading to prolonged hospital stays, extra costs, and disruptions in patient flow. A previous study by Hamid et al. demonstrated that reducing fasting time could arguably mitigate acute kidney injury, avoiding associated extended hospital stays and economic implications (Hamid et al. 2014). Another study revealed that more patients required fluid bolus administration for hypotension in the overnight fasted group than in the limited fasting group (Li et al. 2017).

Additionally, a previous study reported a lower cost of care with the non-fasting approach than with traditional fasting practices (Mishra et al. 2019). Our findings indicate unnecessary implementation of the conventional fasting approach in MICPs. The fact that emergency procedures carry the most risk and are often performed on non-fasting patients supports our findings, as emergency procedures have no reported complication rate when patients are not fasted (Sturdivant et al. 2023).

A significant surge in complications or adverse outcomes did not counterbalance the non-inferiority of non-fasting efficacy and the satisfaction score. When safety outcomes were evaluated, the non-fasting approach demonstrated no significant difference in the incidence of intra- or postoperative complications, including aspiration, postprocedural pneumonia, emergency intubation, nausea/vomiting, anti-emetic use, hypoglycemia, intraoperative fluid provision, contrast-induced acute kidney injury, the need for inotropes, vasopressors, or ventilation, in our analysis. Our findings are consistent with those of a previous study, which suggested that the incidence of adverse outcomes was similar between fasting and non-fasting cohorts (Mishra et al. 2019).

Research in other fields reveals no association between non-fasting and aspiration or vomiting when patients receive conscious sedation. A study by Kwon et al. revealed no significant difference in vomiting or nausea between fasting and non-fasting cohorts, with no cases of pulmonary aspiration among 2554 patients. The incidence of vomiting and nausea is low, at 1.05% (Kwon et al. 2011). Similarly, a systematic review of various procedures involving conscious sedation revealed no episodes of aspiration in nonfasted patients undergoing procedures other than endoscopy (Green et al. 2017). Furthermore, research regarding procedural sedation in the emergency department has not shown any association between fasting duration and the incidence of vomiting or other complications (Taylor et al. 2011; Thorpe and Benger 2010; Wenzel-Smith and Schweitzer 2011). Current guidelines for conscious sedation in the emergency department indicate that fasting is unnecessary as long as verbal communication is maintained ( (Safe Sedation of Adults in the Emergency Department Report and Recommendations by The Royal College of Anaesthetists and The College of Emergency Medicine Working Party on Sedation, Anaesthesia and Airway Management in the Emergency Department, 2012).

### Strengths

The key strength of our analysis is that it is the first meta-analysis evaluating outcomes for non-fasting and traditional fasting protocols in patients undergoing MICPs, incorporating a large sample size without any selection bias associated with the selective publication of results from specialized centers. Including only RCTs ensures that our results reflect the real-world impact of non-fasting rather than traditional fasting in patients undergoing MICPs. Overall, our pooled analysis contributes to a more reliable understanding of the implications of non-fasting protocols in MICPs.

### Limitations

When interpreting the findings of this meta-analysis, it is vital to recognize its limitations. The outcome data were not adjusted based on individual risk profiles, as our analysis did not utilize patient-level data. Additionally, while these findings are compelling, they should be interpreted within the context of the individual patient’s clinical profile and the nature of specific cardiac procedures. The single-blinded design of most RCTs and the subjective nature of patient self-reported satisfaction surveys may limit the robustness of our evidence. Given that the baseline age in all included RCTs was older, the results should be approached with prudence in young patients. Furthermore, most of the studies we pooled in our analysis were conducted in the USA; hence, the results might not represent the global population, and caution should be exercised when using results outside the USA.

### Implications for research, practice, and policy

Considering strong evidence from our meta-analysis, we can confidently suggest that non-fasting protocols for MICPs are a viable and safe alternative to traditional fasting approaches. This highlights the need to revise fasting protocols for MICPs. Furthermore, more high-powered and large-scale RCTs are necessary to validate these findings and explore the benefits of non-fasting protocols in MICPs that aim to establish the best evidence-based clinical practice.

## Conclusion

The non-fasting protocol demonstrates comparable efficacy, safety, and overall satisfaction to the conventional fasting approach. This indicates that non-fasting may be a viable, patient-friendly alternative without compromising clinical outcomes.

## Supplementary Information

Additional file 1: Table S1. Search strategy. Figure S1. Sensitivity analysis of composite satisfaction score. Figure S2. Sensitivity analysis of hunger score. Figure S3: Sensitivity analysis of thirst score. Figure S4. Forest plot of length of hospital stay. Figure S5. Forest plot of post-operative creatinine level. Figure S6. Forest plot of heart rate at procedure start.

## Data Availability

No datasets were generated or analysed during the current study.
