# Supplementary material for: Non-fasting versus fasting before percutaneous cardiac procedures: a systematic review and meta-analysis of randomized controlled trials
Source: Perioper Med (Lond). 2025 Feb 28;14:24. doi: 10.1186/s13741-024-00485-6 (PMC11869692; doi:10.1186/s13741-024-00485-6)
Supplement: Supplementary file 1 — Additional file 1: Table S1. Search strategy. Figure S1. Sensitivity analysis of composite satisfaction score. Figure S2. Sensitivity analysis of hunger score. Figure S3: Sensitivity analysis of thirst score. Figure S4. Forest plot of length of hospital stay. Figure S5. Forest plot of post-operative creatinine level. Figure S6. Forest plot of heart rate at procedure start. [file 13741_2024_485_MOESM1_ESM.docx]

**Title.**

Non-Fasting versus Fasting before Percutaneous Catheterization Cardiac Procedures: A Systematic Review and Meta-Analysis of Randomized Controlled Trials

**Running Title.**

Non-fasting versus fasting in PCI.

**Authors.**

Elsayed Balbaa*^1,8^, Ahmed A. Ibrahim^2^, Mohammad Bazzazeh^1^, Shehroze Tabassum^3^, Shrouk Ramadan^5^, Ahmed Farid Gadelmawla^2,8^, Abdelrahman Elshimy^1^, Obieda Altobaishat^6^, Mohamed Abuelazm^7^

**Affiliations.**

1. Faculty of Medicine, Alexandria University, Alexandria, Egypt

2. Faculty of Medicine, Menoufia University, Menoufia, Egypt

3. Department of Medicine, King Edward Medical University, Lahore, Pakistan

4. Faculty of Medicine, Menoufia University, Menoufia, Egypt

5. Faculty of Medicine, Ain Shams University, Cairo, Egypt

6. Faculty of Medicine, Jordan University of Science and Technology, Irbid, Jordan

7. Faculty of Medicine, Tanta University, Tanta, Egypt

8. Medical Research Group of Egypt (MRGE), Negida Academy, Arlington, MA, USA.

**Corresponding author.**

Elsayed Balbaa

Faculty of Medicine, Alexandria University, Alexandria, Egypt

Medical Research Group of Egypt (MRGE), Negida Academy, Arlington, MA, USA.

**Email:** [elsayedbalbaa.med@gmail.com](mailto:elsayedbalbaa.med@gmail.com)

**Supplementary materials:**

**Content:**

**Tables.**

Table S1: Search strategy.

**Figures.**

Figure S1: Sensitivity analysis of composite satisfaction score.

Figure S2: Sensitivity analysis of hunger score.

Figure S3: Sensitivity analysis of thirst score.

Figure S4: Forest plot of length of hospital stay.

Figure S5: Forest plot of post-operative creatinine level.

Figure S6: Forest plot of heart rate at procedure start.

| Database | Search Terms | Search Field | Search Results |
| --- | --- | --- | --- |
| Pubmed | ((Fasting OR Fast* OR "non per oral" OR "nil per oral" OR "nil per os" OR "NPO" OR "nothing by mouth" OR "nothing per mouth" OR "Oral free" OR "food restriction" OR "Hunger Strike*" OR Famine OR Restricted) AND (liberal OR nonfast* OR "non fast*" OR "non-fast*" OR "Cardiac diet" OR "HEART-HEALTHY DIET" OR feast)) AND ("interventional cardiology" OR "Cardiac intervention" OR "Percutaneous coronary intervention" OR "PCI" OR "Cardiac Catheterization" OR "Cardiac electronic device implantation" OR "Cardiac device implantation" OR "coronary Angiograph*" OR "Pacemaker implantation" OR "cardiac ablation" OR "atrial ablation" OR "cardiac procedure* " OR "TAVR" OR "transcatheter aortic valve replacement" OR "TAVI" OR "transcatheter aortic valve implantation") | All Field | 38 |
| Cochrane | ((Fasting OR Fast* OR "non per oral" OR "nil per oral" OR "nil per os" OR "NPO" OR "nothing by mouth" OR "nothing per mouth" OR "Oral free" OR "food restriction" OR "Hunger Strike*" OR Famine OR Restricted) AND (liberal OR nonfast* OR "non fast*" OR "non-fast*" OR "Cardiac diet" OR "HEART-HEALTHY DIET" OR feast)) AND ("interventional cardiology" OR "Cardiac intervention" OR "Percutaneous coronary intervention" OR "PCI" OR "Cardiac Catheterization" OR "Cardiac electronic device implantation" OR "Cardiac device implantation" OR "coronary Angiograph*" OR "Pacemaker implantation" OR "cardiac ablation" OR "atrial ablation" OR "cardiac procedure* " OR "TAVR" OR "transcatheter aortic valve replacement" OR "TAVI" OR "transcatheter aortic valve implantation") | All Field | 28 |
| WOS | ((Fasting OR Fast* OR "non per oral" OR "nil per oral" OR "nil per os" OR "NPO" OR "nothing by mouth" OR "nothing per mouth" OR "Oral free" OR "food restriction" OR "Hunger Strike*" OR Famine OR Restricted) AND (liberal OR nonfast* OR "non fast*" OR "non-fast*" OR "Cardiac diet" OR "HEART-HEALTHY DIET" OR feast)) AND ("interventional cardiology" OR "Cardiac intervention" OR "Percutaneous coronary intervention" OR "PCI" OR "Cardiac Catheterization" OR "Cardiac electronic device implantation" OR "Cardiac device implantation" OR "coronary Angiograph*" OR "Pacemaker implantation" OR "cardiac ablation" OR "atrial ablation" OR "cardiac procedure* " OR "TAVR" OR "transcatheter aortic valve replacement" OR "TAVI" OR "transcatheter aortic valve implantation") | All Field | 20 |
| SCOPUS | ((Fasting OR Fast* OR "non per oral" OR "nil per oral" OR "nil per os" OR "NPO" OR "nothing by mouth" OR "nothing per mouth" OR "Oral free" OR "food restriction" OR "Hunger Strike*" OR Famine OR Restricted) AND (liberal OR nonfast* OR "non fast*" OR "non-fast*" OR "Cardiac diet" OR "HEART-HEALTHY DIET" OR feast)) AND ("interventional cardiology" OR "Cardiac intervention" OR "Percutaneous coronary intervention" OR "PCI" OR "Cardiac Catheterization" OR "Cardiac electronic device implantation" OR "Cardiac device implantation" OR "coronary Angiograph*" OR "Pacemaker implantation" OR "cardiac ablation" OR "atrial ablation" OR "cardiac procedure* " OR "TAVR" OR "transcatheter aortic valve replacement" OR "TAVI" OR "transcatheter aortic valve implantation") | Title, Abstract, Keywords | 34 |
| EMBASE | ((Fasting OR Fast* OR "non per oral" OR "nil per oral" OR "nil per os" OR "NPO" OR "nothing by mouth" OR "nothing per mouth" OR "Oral free" OR "food restriction" OR "Hunger Strike*" OR Famine OR Restricted) AND (liberal OR nonfast* OR "non fast*" OR "non-fast*" OR "Cardiac diet" OR "HEART-HEALTHY DIET" OR feast)) AND ("interventional cardiology" OR "Cardiac intervention" OR "Percutaneous coronary intervention" OR "PCI" OR "Cardiac Catheterization" OR "Cardiac electronic device implantation" OR "Cardiac device implantation" OR "coronary Angiograph*" OR "Pacemaker implantation" OR "cardiac ablation" OR "atrial ablation" OR "cardiac procedure* " OR "TAVR" OR "transcatheter aortic valve replacement" OR "TAVI" OR "transcatheter aortic valve implantation") | All Field | 43 |

Table S1: Search strategy.


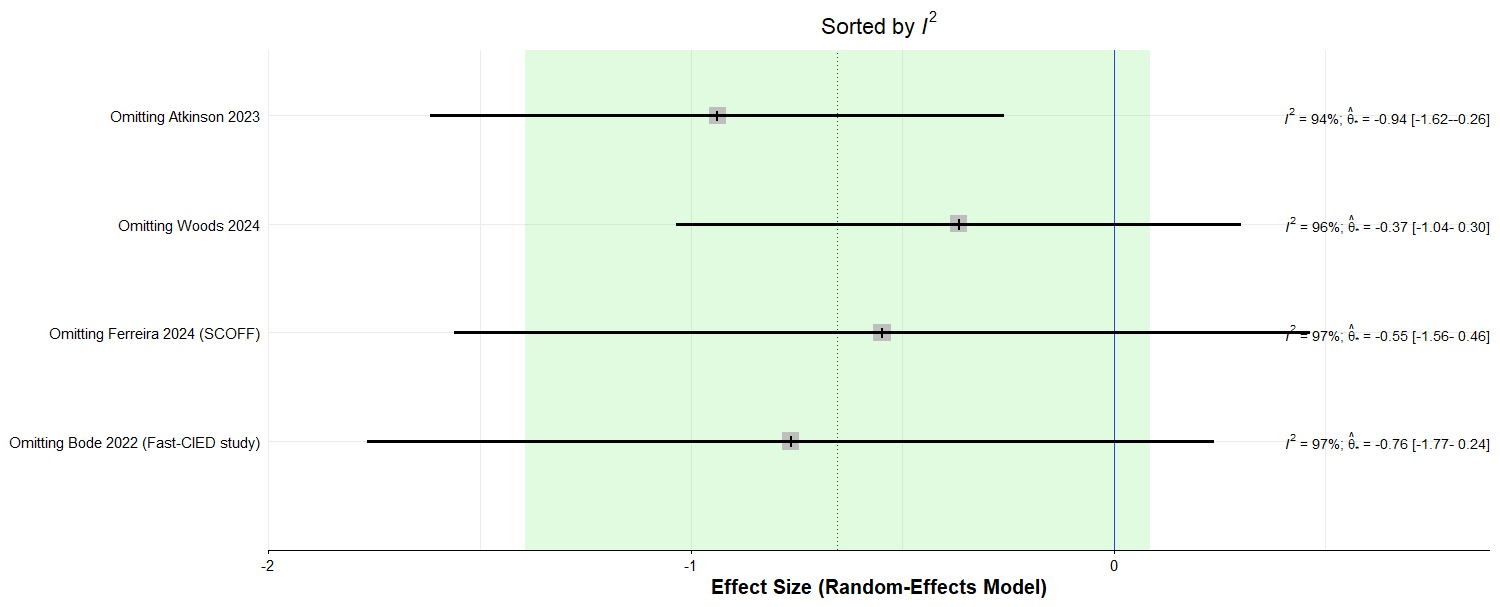


Figure S1: Sensitivity analysis of composite satisfaction score.


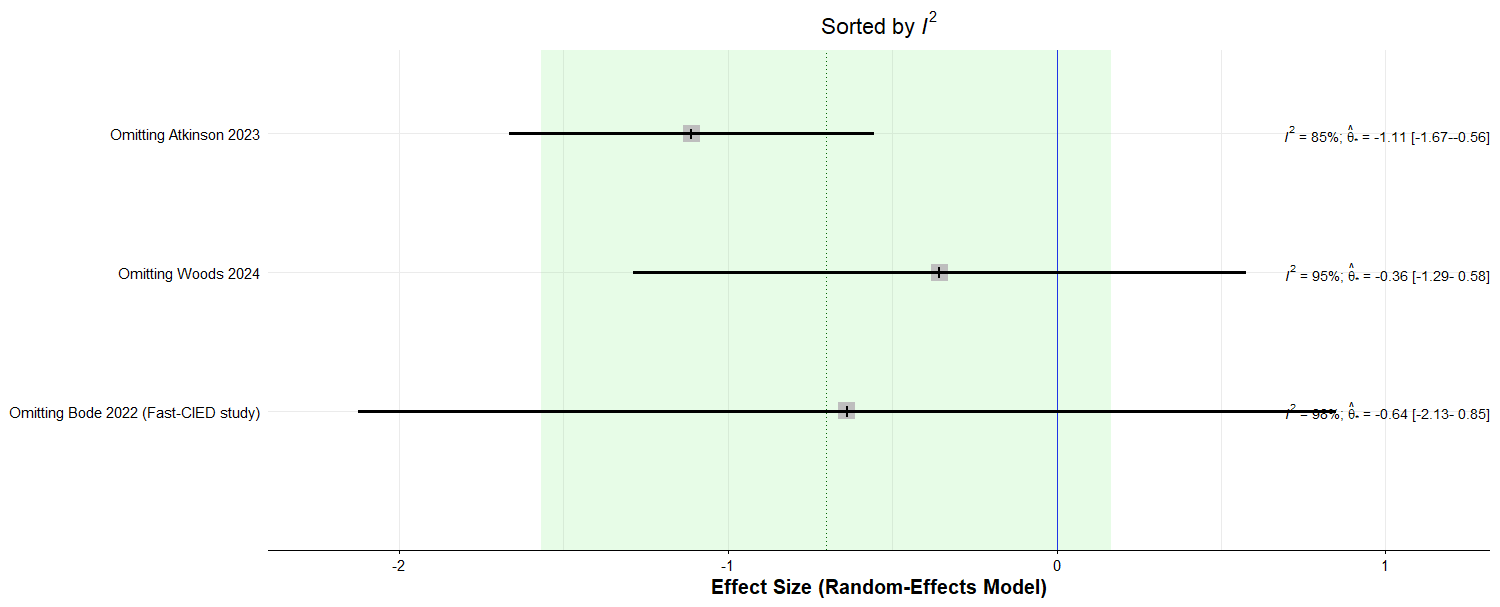


Figure S2: Sensitivity analysis of hunger score.


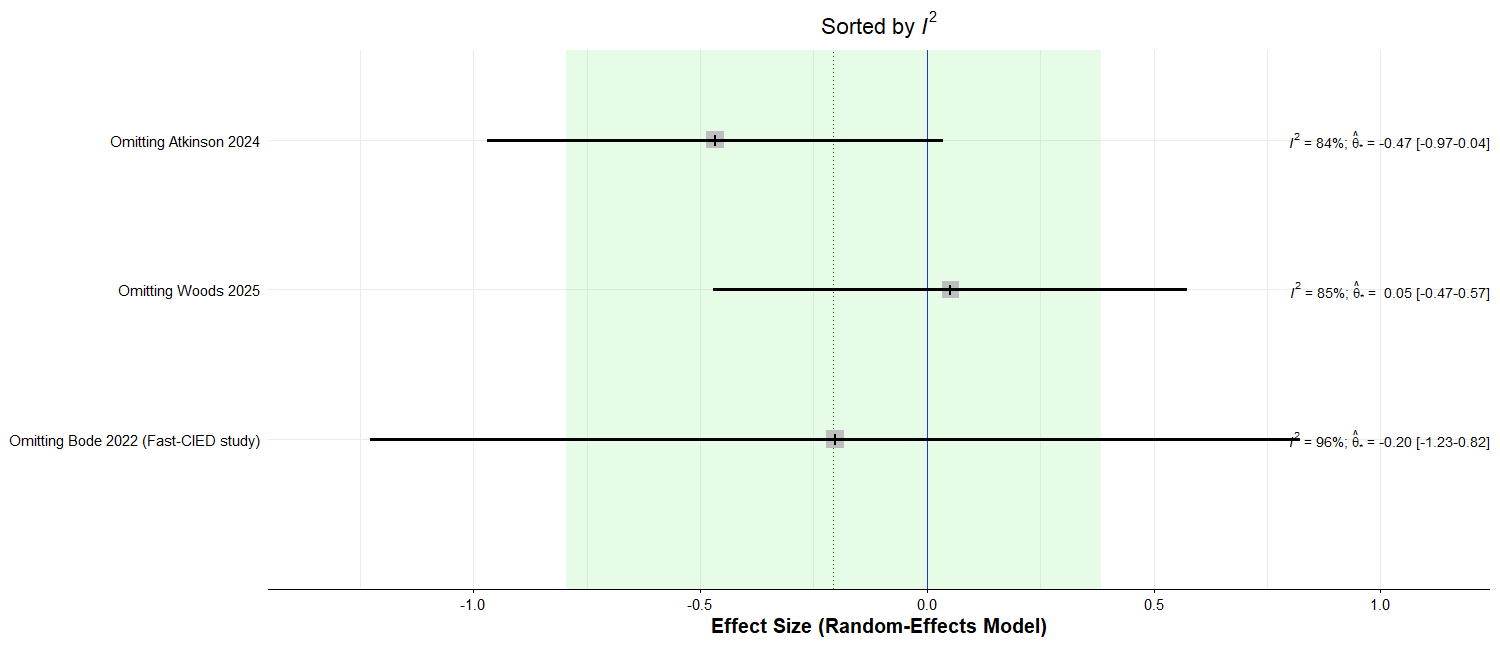


Figure S3: Sensitivity analysis of thirst score.


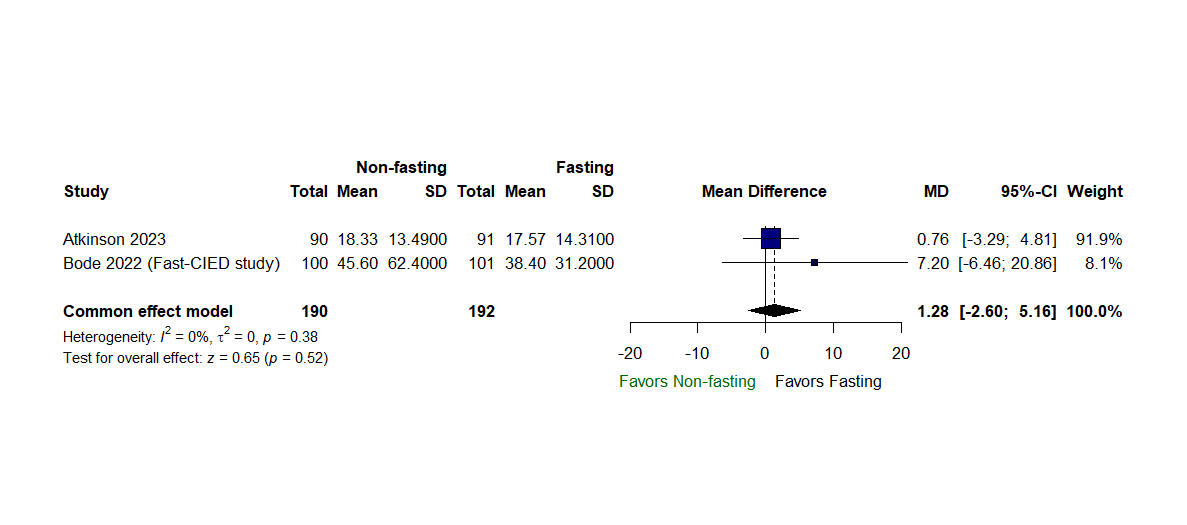


Figure S4: Forest plot of length of hospital stay.


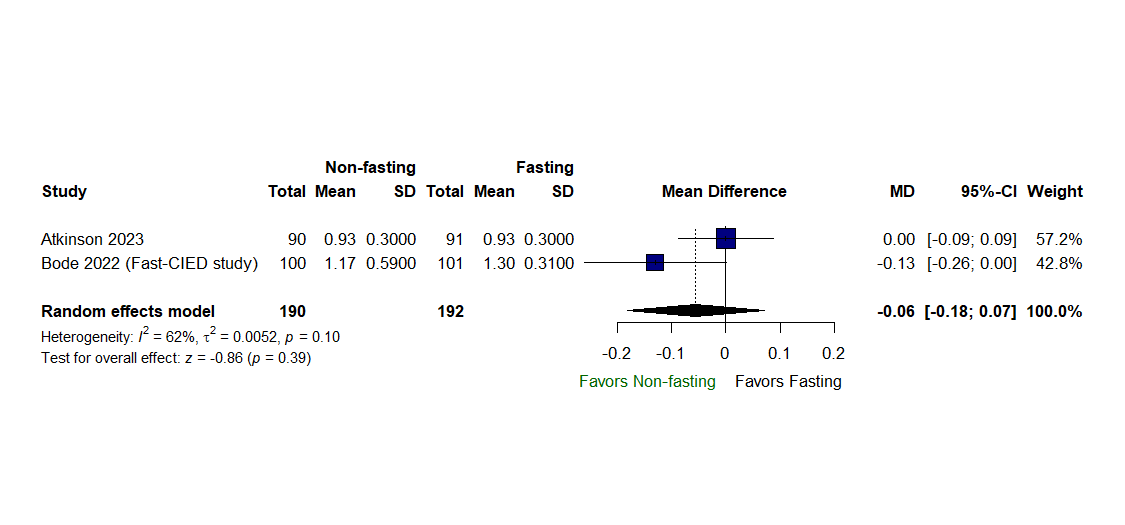


Figure S5: Forest plot of post-operative creatinine level.


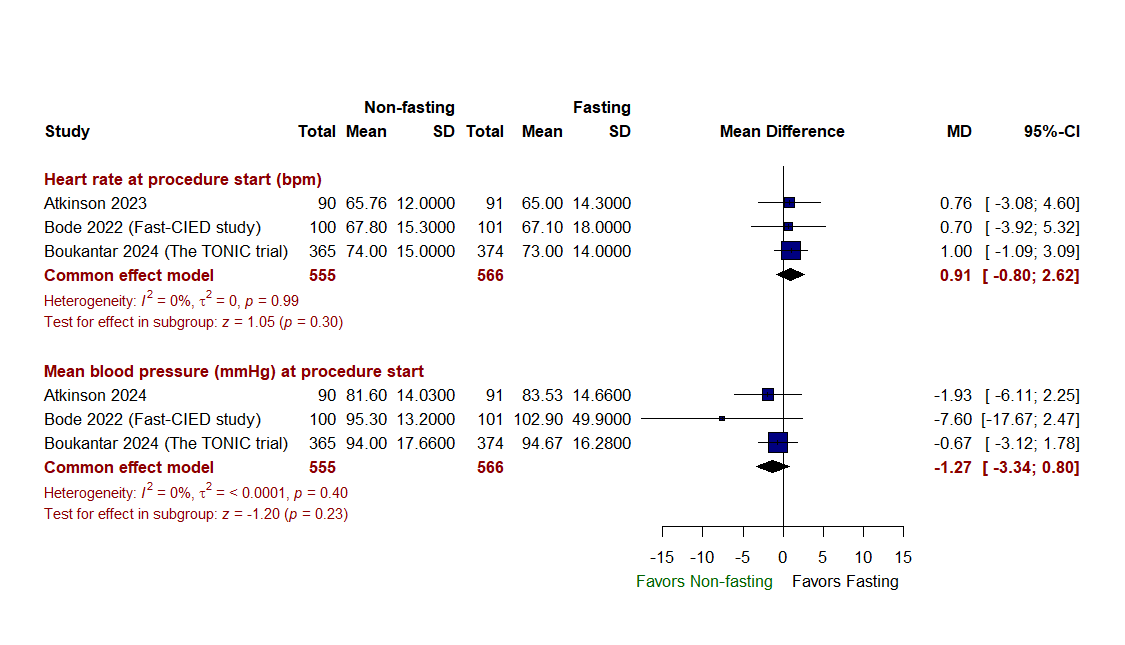


Figure S6: Forest plot of heart rate & mean arterial blood pressure at procedure start.
